# Role of preoperative intravenous iron therapy to correct anemia before major surgery: study protocol for systematic review and meta-analysis

**DOI:** 10.1186/s13643-015-0016-4

**Published:** 2015-03-15

**Authors:** Abdelsalam M Elhenawy, Steven R Meyer, Sean M Bagshaw, Roderick G MacArthur, Linda J Carroll

**Affiliations:** 1 School of Public Health, University of Alberta, 4075 RTF, 8308 114 Street, Edmonton, Alberta T6G 2E1 Canada; 2 Division of Cardiac Surgery, Department of Cardiac Surgery, Faculty of Medicine and Dentistry, University of Alberta, 8440-112 Street, Edmonton, Alberta T6G 2B7 Canada; 3 Division of Critical Care Medicine, Faculty of Medicine and Dentistry, University of Alberta, 2-124 Clinical Sciences Building 8440-112 Street, Edmonton, Alberta T6G 2B7 Canada

**Keywords:** Intravenous iron therapy, Preoperative anemia, Major surgery

## Abstract

**Background:**

Preoperative anemia is a common and potentially serious hematological problem in elective surgery and increases the risk for perioperative red blood cell (RBC) transfusion. Transfusion is associated with postoperative morbidity and mortality. Preoperative intravenous (IV) iron therapy has been proposed as an intervention to reduce perioperative transfusion; however, studies are generally small, limited, and inconclusive.

**Methods/Design:**

We propose performing a systematic review and meta-analysis. We will search MEDLINE, EMBASE, EBM Reviews, Cochrane-controlled trial registry, Scopus, registries of health technology assessment and clinical trials, Web of Science, ProQuest Dissertations and Theses, and conference proceedings in transfusion, hematology, and surgery. We will contact our study drug manufacturer for unpublished trials. Titles and abstracts will be identified and assessed by two reviewers for potential relevance. Eligible studies are: randomized or quasi-randomized clinical trials comparing preoperative administration of IV iron with placebo or standard of care to reduce perioperative blood transfusion in anemic patients undergoing major surgery. Screening, data extraction, and quality appraisal will be conducted independently by two authors. Data will be presented in evidence tables and in meta-analytic forest plots.

Primary efficacy outcomes are change in hemoglobin concentration and proportion of patients requiring RBC transfusion. Secondary outcomes include number of units of blood or blood products transfused perioperatively, transfusion-related acute lung injury, neurologic complications, adverse events, postoperative infections, cardiopulmonary complications, intensive care unit (ICU) admission/readmission, length of hospital stay, acute kidney injury, and mortality.

Dichotomous outcomes will be reported as pooled relative risks and 95% confidence intervals. Continuous outcomes will be reported using calculated weighted mean differences. Meta-regression will be performed to evaluate the impact of potential confounding variables on study effect estimates.

**Discussion:**

Reducing unnecessary RBC transfusions in perioperative medicine is a clinical priority. This involves the identification of patients at risk of receiving transfusions along with blood conservation strategies. Of potential pharmacological blood conservation strategies, IV iron is a compelling intervention to treat preoperative anemia; however, existing data are uncertain. We propose performing a systematic review and meta-analysis evaluating the efficacy and safety of IV iron administration to anemic patients undergoing major surgery to reduce transfusion and perioperative morbidity and mortality.

**Systematic review registration:**

PROSPERO CRD42015016771

**Electronic supplementary material:**

The online version of this article (doi:10.1186/s13643-015-0016-4) contains supplementary material, which is available to authorized users.

## Background

### Epidemiology of preoperative anemia

Anemia is defined by the World Health Organization (WHO) as a hemoglobin concentration less than 13 g/dL in men and 12 g/dL in women [1]. Preoperative anemia is the most common hematological abnormality among the patients undergoing major elective surgery [2]. The prevalence of preoperative anemia ranges from 5% to 75%, depending on patient susceptibilities and the proposed surgical procedure [3]. Preoperative anemia is more common among older patients and those with chronic disease such as heart failure, diabetes mellitus, chronic kidney disease, primary hematologic diseases, other inflammatory diseases [4], and coronary artery disease [5,6]. Although diagnosis and treatment of anemia preoperatively is essential to optimize the patient’s condition [7], preoperative anemia treatment is not a priority for most surgeons [8].

### Epidemiology of blood transfusion and outcomes

Preoperative anemia has been associated with an increased risk for 30-day mortality [9,10]. Preoperative anemia has also been shown to be an independent risk factor for perioperative red blood cell (RBC) transfusion and for postoperative morbidity [11]. Major morbid outcomes include transfusion-related acute lung injury (TRALI) [12], nosocomial infections [13], increased graft occlusion after coronary artery bypass grafting (CABG) [14], myocardial events, neurological events, acute kidney injury [15], tumor recurrence [16], and suppressed immune function [17]. In a recent large study of 22,785 consecutive patients, investigators found that transfusing as little as 1 or 2 units of RBCs was associated with increased morbidity and mortality after cardiac surgery [18] supporting a previous systematic review of observational studies [19]. The findings from these studies would imply that strategies to reduce unnecessary RBC transfusions might be associated with improved postoperative outcomes.

RBC transfusions are also associated with significant cost related both to the product itself and the morbid events associated with unnecessary RBC transfusions which contribute to additional direct and indirect hospitalization costs [20]. In a recent RBC transfusion cost analysis study, the actual cost for each RBC unit was reported to range between US $522 and US $1,183 after calculating the direct and indirect costs, which include consumables, laboratory testing, nursing time, patient transport, treatment costs, and staff fees resulting in increased cumulative total costs [21]. This is substantially higher than previous estimates of the cost of each RBC unit at US $250 to $550 [22].

National USA data [23] showed a decline in blood transfusion usage estimate by 3% over each of the 2 years (2009 to 2010), and similar data [24] have been reported in the UK as well. Although many therapeutic modalities have been initiated to minimize the patients’ requirement for perioperative RBC transfusion, the rate of transfusion remains unacceptably high and variable across both cardiac (17 to 80%) [25,26] and non-cardiac major surgery [27]. These observations would imply that transfusion practices are variable across patient, provider, and health system factors. This may stem from the absence of high-quality evidence to guide the management of perioperative anemia and blood conservation.

### Strategies for perioperative blood conservation

A variety of contemporary perioperative pharmacologic and non-pharmacologic blood conservation strategies (Table 1) [28] have been proposed and variably adopted to minimize RBC transfusion. In a recent study, the outcomes of 322 Jehovah’s Witness (JW) patients undergoing cardiac surgery, who, for personal beliefs, refuse transfusion of all blood products including RBCs, were evaluated. In this study, JW patients had comparable long-term survival to those willing to receive RBC transfusion in a propensity-matched analysis; however, JW patients had fewer postoperative complications and shorter intensive care unit (ICU) lengths of stay [29]. These provocative findings would imply that many RBC transfusions might be unnecessary. In fact, these data suggest that blood conservation strategies, including preoperative treatment of anemia, may either reduce exposure to RBC transfusions or even act as a trigger for RBC transfusion. In addition, a more recent meta-analysis of randomized trials with 8,735 patients showed lower risk of health care-associated infection for a restrictive RBC transfusion strategy in comparison to a liberal transfusion strategy in hospitalized patients [30].

**Table 1:** **Current strategies for perioperative blood conservation**

Conservation strategy	Examples
1. Pharmacological therapies	a. Antifibrinolytic drugs (aprotinin, tranexamic acid, epsilon-aminocaproic acid)
	b. Desmopressin acetate
	c. Recombinant factor VIIa (rFVIIa)
	d. Erythropoietin (EPO)
	e. Topical haemostatic agents
2. Autologous blood transfusion	a. Preoperative autologous blood donation
	b. Acute normovolemic hemodilution
	c. Red cell salvage (intraoperative and postoperative)
3. Anesthetic techniques	a. Controlled hypotension
	b. Spinal or epidural anesthesia
	c. Central venous pressure (CVP) manipulation
4. Surgical techniques	a. Coagulation diathermy devices, lasers, and ultrasonic scalpels
	b. Minimally invasive surgery
	c. Endoscopic and laparoscopic surgery
5. Blood substitutes	a. Solutions of modified hemoglobin
	b. Perfluorocarbon emulsions
6. Transfusion protocols, guidelines, and clinical audit	

### Preoperative intervention: prophylactic iron therapy

Preoperative pharmacologic treatment of anemia has been proposed to reduce perioperative RBC transfusion [31,32]. One potential strategy is the use of intravenous (IV) iron. Iron is fundamental in RBC formation and is the most common nutritional deficiency in both developed and developing countries [33]. A recent study [34] reported that usage of oral iron to treat iron deficiency anemia is limited by gastrointestinal absorption, particularly, in the patients with associated acute or chronic diseases [35]. IV iron has been reported to increase the hemoglobin (Hb) level and to replenish iron stores more rapidly than oral iron formulation in women with post-partum iron deficiency anemia in a variety of RCT studies in other areas of medicine such as post-partum hemorrhage [36], but in surgery, most have been observational in nature. Consequently, preoperative IV iron has been proposed as an efficacious strategy to increase serum hemoglobin and minimize exposure to perioperative RBC transfusions [37,38].

To date, there is no broad consensus or established clinical practice guideline to support the routine use of prophylactic IV iron to treat anemia before major elective surgery. Moreover, additional questions about the optimal timing of therapy, dose of iron, and whether anemic patients need additional nutritional supplementation have yet to be answered. However, there are a few guidelines recommending the use of IV for anemia in surgical cases, but all are lacking class 1A evidence-based medicine [39-41]. To date, there are relatively few studies, the majority of which have been small and non-definitive, that have evaluated the efficacy and safety of IV iron preparations in patients undergoing major elective surgery. A recent meta-analysis [42] showed that very low-quality evidence of IV iron results in modest increases in hemoglobin levels compared with oral iron or inactive control, but without clinical benefit. Unfortunately, this meta-analysis was less homogeneous than would be ideal, and they were not able to do subgroup analyses for different types of participants (blood loss, cancer, preoperative anemia, chronic heart failure, autoimmune disorders, and infectious disease). In addition, their literature search ended July 2013. Three other recent studies have been published as well. The first is a systematic review for RCTs with restricted language and search time frame [43], the second is a systematic review and meta-analysis for RCTs restricted to colorectal surgery [44], and the third is a systematic literature review of RCTs and observational studies restricted to cardiac surgery [45]. All three failed to find a sufficient evidence to support the use of IV iron to decrease RBC transfusion.

Accordingly, we propose to perform a systematic review and evidence synthesis on the efficacy and safety of IV iron therapy in the preoperative setting to treat anemia, reduce transfusions, and improve outcome. This review will capture recently published studies, will include a broad range of elective surgery, and will not be limited to English publications or RCTs.

### Hypothesis

We hypothesize preoperative IV iron therapy will improve preoperative hemoglobin concentrations in anemic patients undergoing major elective surgery, reduce the need for RBC transfusion, and reduce complications compared with placebo or standard of care. We will synthesize the available data on the efficacy and safety of IV iron therapy to increase hemoglobin levels, avoid transfusions, and improve outcomes for anemic patients undergoing major elective surgery.

### Objectives

Perform a systematic review and evidence synthesis of all randomized and quasi-randomized studies investigating (a) the efficacy of IV iron administration to improve preoperative hemoglobin concentration and reduce RBC transfusion rate, (b) the safety of IV iron formulations with respect to adverse effects, and (c) the effectiveness of IV iron administration to reduce perioperative major morbidity and health resource use.

## Methods/Design

### Search strategy

In consultation with a health sciences librarian at the John W. Scott Health Science Library at the University of Alberta, we will search MEDLINE, EMBASE, EBM Reviews, and the Cochrane-controlled trial registry in the Cochrane library, Scopus, registries of health technology assessment and clinical trials, and Web of Science. We will search the literature using the following search terms: iron or dextran or Venofer or ferric or ferrous or ferrlecit AND anemi* or anaemi* AND preoperat* or postoperat* or perioperat* or operati* or surg* or presurg* or postsurg* or perisurg* AND random* or trial or placebo*. An example of the search conducted in MEDLINE is in Additional file 1.

In addition, we will contact our study drug manufacturer for unpublished trials, and we will search the ProQuest Dissertations and Theses database. Selected conference proceedings in transfusion, hematology, and surgery will also be searched.

We will start from the earliest retrievable date of each database to February 2015, supplemented by a manual search of reference lists of retrieved trials. In addition, reference lists of prior reviews of similar topics will be searched for relevant studies. Language will be unrestricted.

### Inclusion criteria

All the included articles have to fulfill these criteria:

▪ Designs included are randomized and quasi-randomized studies in all different phases.
▪ Study compares any type of intravenous iron administered preoperatively to placebo or standard care.
▪ Study reports findings specific to adult humans.
▪ Study of patients undergoing any elective major surgery (Additional file 2) including, but not limited to, cardiac, thoracic, orthopedic, gastrointestinal, brain, urological, or obstetric operations.
▪ Study patients have pre-treatment hemoglobin level less than 12 g/dL.
▪ Study reports at least one of the two following outcomes: absolute or relative change in preoperative hemoglobin level and/or the proportion of patients receiving perioperative allogeneic RBC transfusion.

### Exclusion criteria

Any one of these criteria will result in a study being excluded:

▪ Observational (non-experimental) studies, reviews, opinion papers, letters to the editor, and studies with no reported methodology.
▪ Studies with no adult-specific findings.
▪ Studies involving patients with pre-treatment hemoglobin level greater than 12 g/dL.
▪ Studies using oral iron only.
▪ Studies using IV iron plus EPO.
▪ Studies of minimally invasive robotic and laparoscopic surgery.

#### Primary outcomes

Co-primary outcomes:

▪ Absolute and relative change in preoperative hemoglobin concentration.
▪ Proportion of anemic patients who receive allogeneic RBC transfusion at any time perioperatively.

#### Secondary outcomes

These will focus mainly on safety-related outcomes:

▪ Total number of units of blood or blood products transfused perioperatively.
▪ All-cause mortality.
▪ Postoperative nosocomial infection (Additional file 2).
▪ TRALI (Additional file 2).
▪ Neurologic complications (Additional file 2).
▪ Acute kidney injury (Additional file 2).
▪ Any reported adverse reaction (Additional file 2).
▪ Any reported reaction or side effect (Additional file 2) from receiving a RBC transfusion. These may include, but are not limited to, hemolysis of transfused red cells, alloimmunization, development of antibodies against platelets or white blood cells, post-transfusion purpura, graft *vs.* host disease, infection; immunomodulation, and iron overload.
▪ Serious adverse events (SAEs), including suspected and serious unexpected serious adverse reactions (SUSARs) (Additional file 2).
▪ ICU admission and/or readmission and length of hospital stay.

### Study screening

Two authors (AE and SM) will initially review titles and abstracts to retrieve potentially relevant studies. Retrieved studies will then be subjected to a second phase of screening for eligibility, as determined by the eligibility criteria listed above. Reason(s) for ineligibility will be documented for all studies excluded in the second phase of screening, using pre-piloted forms. Disagreements will be resolved through discussion or by a third reviewer (SB) if necessary. We will provide the PRISMA study flow chart (Additional file 3).

### Data abstraction

Data will be abstracted from the reports of all the included studies in duplicate and independently by two reviewers (AE and SM) on standardized and pre-piloted data extraction forms (Additional file 4). For the studies evaluating multiple treatment arms, comparisons will be made between IV iron arm and standard care or placebo arm only. Discrepancies in extracted data will be resolved by consensus, or involving a third author (SB) where consensus cannot be reached.

Abstracted data from each study will include the details on the following:

- Study design, methodology, analysis, funding source, trial registration, and publication details.
- Aggregate participant demographic characteristics (for example, age, sex, and race).
- Aggregate participant characteristics including comorbid diseases and risk factors for anemia (for example, diet, any cause of blood loss, chronic or serious illnesses or infections, a family history of anemia, reasons/indications for major surgery).
- Dosage of drug administered, frequency, and duration of use.
- Hematocrit monitoring.
- Iron-related blood tests including serum iron, ferritin, transferrin, total iron-binding capacity (TIBC), transferrin saturation (iron saturation of transferrin), and unsaturated iron-binding capacity (UIBC).
- All primary and secondary outcomes reported.
- Study quality features (see below).

### Assessment of methodological quality

To evaluate the risk of bias in both RCTs and quasi-randomized trials, two independent reviewers will use the Cochrane Collaboration’s tool for assessing risk of bias. This tool provides a model to assess the following domains of bias: random sequence generation, allocation concealment, blinding of participants and personnel, blinding of outcome assessment, incompleteness of outcome data (attrition bias), blinding of outcome assessment (performance and detection bias), selective outcome reporting (reporting bias), and *a priori*-derived sample size calculations. Each domain for each trial will have a ranking of “low,” “unclear,” or “high” risk of bias, in accordance with the Cochrane Collaboration’s approach (Additional file 5) [46].

## Study synthesis plan

### Analysis plan

We will report the results of our search in a PRISMA flow chart, including the number of randomized and quasi-randomized studies (with their citations), the number of phase I, II, and III trials, and the number addressing our primary and secondary outcomes and language of studies. We will present tables outlining a) each study’s characteristics, b) risk of bias for each study, and c) study results. Randomized studies and quasi-randomized studies will be presented in separate tables, and within each set of tables, there will be further stratification by a) outcome addressed (primary *versus* secondary), b) study phase, and c) high, medium, and low risk of bias. This information will also be summarized in the text and the main biases identified for each study design and outcome.

The tables describing study characteristics will include information abstracted from the studies as per the data abstraction section above. A separate table will report each study’s effect measures (findings) corresponding to our primary and secondary outcomes as follows: risk ratios (and their 95% confidence intervals) reflecting the association between preoperative IV iron administration and need for RBC transfusion, differences in change in hemoglobin concentration (and statistical significant of this difference) between those who do and do not receive IV iron, proportion and relative risk of unplanned ICU admissions and of ICU readmissions in each group, differences in hospital length of stay, and 30-day mortality in each group.

### Meta-analysis

We will examine clinical homogeneity of the studies first, followed by assessment of statistical homogeneity by using Cochrane’s *Q* test and *I*^2^ statistics, with *I*^2^ > 40% considered significant heterogeneity [47]. We anticipate that there will be sufficient homogeneity across studies to justify a pooled statistical synthesis; however, we will conduct a qualitative synthesis if there is statistical heterogeneity. We consider the factors listed below as potential sources of heterogeneity in studies.

Data from all trials fulfilling the eligibility criteria will be pooled for meta-analysis using a random effects model to accommodate the anticipated heterogeneity among study results and assuming that the individual specific effects are uncorrelated with the exposure variables [48]. Weights will be assigned to reflect sample size differences. For continuous outcomes (change in Hb concentration, the number of transfused RBC among those transfused, ICU, and hospital lengths of stay), we will calculate the standardized mean difference (SMD) and test for group differences using unpaired *t*-tests of Mann-Whitney as appropriate with *P* < 0.05 signifying statistical significance. The SMD is used as a summary effect size, anticipating the included trials all assessing the same outcome. For categorical outcomes (nosocomial infection, TRALI, neurological complications, acute kidney injury, adverse reactions, ICU admission and readmission), we will calculate pooled relative risks (RR) with 95% confidence intervals. Where studies report length of stay and 30-day mortality using time to event analyses, we will calculate pooled hazard ratios with 95% confidence intervals. The meta-analysis will be performed in RevMan Version 5.3 software [49] and Stata V.13 (STATA Corp, College Station, TX, USA) [50].

### Sensitivity analysis, subgroup analysis, and meta-regression analysis

We will conduct sensitivity analyses to assess the impact of each trial on the overall results and to examine whether an individual study is over-influencing the meta-analysis result. We will do an additional sensitivity analyses by excluding studies with unclear or inadequate randomization. With availability of at least three trials, we will conduct the following subgroup analyses to explore heterogeneity and to assess robustness of our results:

1. Cardiac *versus* non-cardiac;
2. Different IV iron preparations;
3. Hb level; trials with a mean Hb equal to or greater than 10 g/dL;
4. Age; studies with a mean age over the age of 65;
5. Excluding studies considered at high risk of bias according to the Cochrane risk of bias tool.

Our ability to conduct this subgroup analysis will depend on the information provided in the relevant studies. We will use univariable and bivariable meta-regression analyses to explore the impact of the following variables in each study on the pooled effect estimates for the primary outcome: age, sex, baseline ferritin level, baseline hemoglobin level, baseline hematocrit value, total IV iron dose administered, and the rate of patients requiring allogeneic blood transfusion during or after the surgery. The effect of each variable on the pooled effect size will be considered significant when the *P* value of the change in the estimate is less than 0.05 or when the 95% confidence intervals of the two analyses do not overlap.

### Assessment of publication biases

To identify possible publication bias, we will construct a funnel plot of effect size against the inverse of standard error in the studies. Deviation from this funnel may suggest publication bias [51].

### Grading the strength of the evidence

We will assess the overall quality of the evidence for each outcome in the included trials using the Grading of Recommendations Assessment, Development, and Evaluation (GRADE) approach [52].

### Ethical issues

For our meta-analysis, we will not require a health research ethics board review as all data will be sourced from existing literature.

## Discussion

Anemia is common in patients undergoing major surgery [2]. Over the past two decades, a variety of contemporary pharmacologic blood conservation strategies have been adopted to address preoperative anemia in attempts to minimize unnecessary RBC transfusion. However, the optimal treatment is not yet known due to paucity of the randomized trials, most of which are small and have negative results, which may be a result of being underpowered to detect a treatment effect. Consequently, current knowledge implementation has been suboptimal, and there are no high-quality available clinical practice guidelines to inform best practice. A meta-analysis conducted by pooling these studies may provide new and clinically useful information.

In attempts to minimize the impact of blood loss at the time of surgery, and consequently to avoid RBC transfusion, many treatment modalities have been tried, from advanced techniques in operative procedure and anesthesia alongside with newer drugs to stimulate hematopoiesis to replacing blood by transfusion. Although a large systematic review has shown that antifibrinolytic drugs reduce blood loss and consequently the rate of RBC transfusion [53,54], it would appear that these drugs, while reducing blood loss, are associated with a hypercoagulable state [55]. Similarly, thrombotic events are reported with the erythropoiesis-stimulating agents [56]. Iron plays an essential role in erythropoiesis and is a fundamental component in RBC formation [57]. Thus, use of iron is a compelling potential intervention to treat preoperative anemia. However, existing evidence is uncertain [58,59]. In non-surgical settings, it has been shown that supplementation with IV iron usually results in higher hemoglobin values [60]. Previously used intravenous iron preparations such as iron sucrose (saccharate) and iron dextran have been associated with side effects [61] and anaphylactic reactions [62]. However, newer intravenous preparations, such as Venofer® (iron (III)-hydroxide sucrose), have shown better tolerability [63].

We propose to perform a systematic literature review and meta-analysis evaluating the efficacy and safety of IV iron administration to anemic patients undergoing major surgery for reduce transfusions and perioperative morbidity and mortality.

We hypothesize that IV iron is a safe and effective way to treat preoperative anemia. We further hypothesize that preoperative administration of IV iron will increase the hemoglobin in anemic patients at risk for perioperative RBC transfusion and reduce perioperative allogeneic RBC transfusion, reduce major morbidity, and utilize fewer health resources.

### Strengths of our meta-analysis

- It will be a comprehensive search without restriction for language and time frame.
- It will include only experimental designs (RCTs and quasi-randomized studies) to avoid selection bias.
- We will do critical appraisals of methodological quality for the RCT enabling us to determine whether the pooled findings are affected by study quality.

### Limitations

Our meta-analysis might have the same unintentional biases as any meta-analysis, and these potential biases include

1. Study selection process may inadvertently exclude relevant studies.
2. Standard methods used to conduct meta-analyses may introduce bias if studies are not sufficiently homogeneous.
3. Insufficient quantity or poor quality of the included studies.

### Future research and policy implications of our meta-analysis

These will be guided by our final results. We expect that this study will provide the impetus for future large-scale RCTs in this field.

## Additional files

Additional file 1: **Search strategy.** Sample search strategy for Medline search.
Additional file 2: **Definitions.** Definitions of terms used in the report.
Additional file 3: **PRISMA study flow chart.** PRISMA flow chart detailing included and excluded articles at each screening stage.
Additional file 4: **Data extraction forms.** Forms used to extract data from studies.
Additional file 5: **Risk of bias detection.** Cochrane risk of bias tool.

## Abbreviations

CIs: confidence intervals
ESA: erythropoiesis-stimulating agents
GRADE: Grading of Recommendations Assessment, Development, and Evaluation
Hb: hemoglobin
IV: intravenous
RBCs: red blood cells
RR: relative risks
SAEs: serious adverse events
SAR: serious adverse reaction
SUSARs: suspected and unexpected serious adverse reactions
TSAT: transferrin saturation
TRALI: transfusion-related acute lung injury
WHO: World Health Organization
