# Supplementary material for: Role of preoperative intravenous iron therapy to correct anemia before major surgery: study protocol for systematic review and meta-analysis
Source: Syst Rev. 2015 Mar 15;4:29. doi: 10.1186/s13643-015-0016-4 (PMC4369835; doi:10.1186/s13643-015-0016-4)
Supplement: Additional file 1: — Search strategy. Sample search strategy for Medline search. [file 13643_2015_16_MOESM1_ESM.doc]

Additional file 1: Sample Search Strategy

MEDLINE through OVID search strategy

Database: Ovid MEDLINE(R) In-Process & Other Non-Indexed Citations, Ovid MEDLINE(R) Daily and Ovid MEDLINE(R) <1946 to Present> May 15, 2014

1. Iron/

2. exp Iron Compounds/

3. (iron or dextran or venofer or ferric or ferrous or ferrlecit).mp. [mp=title, abstract, original title, name of substance word, subject heading word, keyword heading word, protocol supplementary concept, rare disease supplementary concept, unique identifier]

4. 1 or 2 or 3

5. exp Anemia/

6. (anemi* or anaemi*).mp. [mp=title, abstract, original title, name of substance word, subject heading word, keyword heading word, protocol supplementary concept, rare disease supplementary concept, unique identifier]

7. 5 or 6

8. 4 and 7

9. exp perioperative care/ or exp perioperative period/

10. exp Specialties, Surgical/

11. exp Surgical Procedures, Operative/

12. (preoperat* or postoperat* or perioperat* or operati* or surg* or presurg* or postsurg* or perisurg*).mp. [mp=title, abstract, original title, name of substance word, subject heading word, keyword heading word, protocol supplementary concept, rare disease supplementary concept, unique identifier]

13. 9 or 10 or 11 or 12

14. 8 and 13

15. randomized controlled trial.pt.

16. clinical trial.pt.

17. randomi?ed.ti,ab.

18. placebo.ti,ab.

19. dt.fs.

20. randomly.ti,ab.

21. trial.ti,ab.

22. groups.ti,ab.

23. or/15-22

24. animals/

25. humans/

26. 24 not (24 and 25)

27. 23 not 26

28. 14 and 27
