# Supplementary material for: Role of preoperative intravenous iron therapy to correct anemia before major surgery: study protocol for systematic review and meta-analysis
Source: Syst Rev. 2015 Mar 15;4:29. doi: 10.1186/s13643-015-0016-4 (PMC4369835; doi:10.1186/s13643-015-0016-4)
Supplement: Additional file 2: — Definitions. Definitions of terms used in the report. [file 13643_2015_16_MOESM2_ESM.docx]

**Definitions**

- Major surgery is defined as any invasive surgical procedure in which an extensive resection is performed, organs are removed, a body cavity is entered or normal anatomy is altered.
- Post-operative nosocomial infection defined as having temperature >38°C in the last 24 hours with signs indicating infection and/or receiving antimicrobials beyond those used for routine perioperative prophylaxis.
- Transfusion-related acute lung injury (TRALI), defined as a syndrome of acute respiratory distress occurring early (generally within 6 hours) following transfusion of allogeneic blood in the absence of pre-existing lung injury and other temporally associated risk factors for acute lung injury.
- Neurologic complications, defined as any focal neurological deficit including stroke, transient ischemic attack or seizure, and/or having an impaired level of consciousness due to delirium, confusion, or coma.
- Acute kidney injury, defined as presence of oliguria (urine output less than 500 ml/24 hours) and/or an increase in serum creatinine level greater than 25% or 44.2 µmol/L from a stable pre-operative baseline level.
- Any reported adverse reaction, defined as any harmful effect potentially attributable to the use of IV iron and requiring specific treatment, and/or change of the dosage regimen, and/or which warrants prevention or withdrawal of the drug.
- Any reported reaction or side effect from receiving a RBC transfusion. These may include, but are not limited to: hemolysis of transfused red cells; alloimmunization; development of antibodies against platelets or white blood cells; post-transfusion purpura; graft vs. host disease; infection, immunomodulation; iron overload.
- An unexpected adverse reaction is any adverse reaction to IV iron with different severity or nature not reported in the information about IV iron either in marketing summary of product, previous literature, or in the investigator's trial protocol. Serious adverse events (SAEs), is an expected or unexpected adverse event defined as a noxious and unintended response to IV iron administration occurring at any dose that prolongs current hospital stay or requires new hospital admission, results in significant disability, cause congenital malformation, or death. Serious adverse reactions (SUSARs) is an adverse reaction that meets the definition of a serious adverse event/reaction not consistent with approved product information.
