# Supplementary material for: Role of preoperative intravenous iron therapy to correct anemia before major surgery: study protocol for systematic review and meta-analysis
Source: Syst Rev. 2015 Mar 15;4:29. doi: 10.1186/s13643-015-0016-4 (PMC4369835; doi:10.1186/s13643-015-0016-4)
Supplement: Additional file 4: — Data extraction forms. Forms used to extract data from studies. [file 13643_2015_16_MOESM4_ESM.doc]

**Additional File 4-A: Description of Included Randomized Clinical Trials.**

| Study ID | Study Author | Year | Number of Patients Randomized | Age (y) Mean ± SD | Type of Surgery | Treatment Arm ( IV Iron Type) | Dosing  Schedule | Control Arm (Placebo/  Standard of Care) | ESA Type and Dosing | Follow Up in Weeks |
| --- | --- | --- | --- | --- | --- | --- | --- | --- | --- | --- |
|  |  |  |  |  |  |  |  |  |  |  |
|  |  |  |  |  |  |  |  |  |  |  |
|  |  |  |  |  |  |  |  |  |  |  |
|  |  |  |  |  |  |  |  |  |  |  |
|  |  |  |  |  |  |  |  |  |  |  |
|  |  |  |  |  |  |  |  |  |  |  |
|  |  |  |  |  |  |  |  |  |  |  |
|  |  |  |  |  |  |  |  |  |  |  |
|  |  |  |  |  |  |  |  |  |  |  |
|  |  |  |  |  |  |  |  |  |  |  |
|  |  |  |  |  |  |  |  |  |  |  |
|  |  |  |  |  |  |  |  |  |  |  |
|  |  |  |  |  |  |  |  |  |  |  |
|  |  |  |  |  |  |  |  |  |  |  |
|  |  |  |  |  |  |  |  |  |  |  |
|  |  |  |  |  |  |  |  |  |  |  |
|  |  |  |  |  |  |  |  |  |  |  |
|  |  |  |  |  |  |  |  |  |  |  |
|  |  |  |  |  |  |  |  |  |  |  |

**Additional File 4-B: Baseline Characteristics of Included Studies**

| Female Sex | Race | Comorbid Disease | Chronic or Serious Illnesses | Nutritional  Deficiency | Chronic Infection | Any Cause of Blood Loss, | Family History of Anemia |
| --- | --- | --- | --- | --- | --- | --- | --- |
|  |  |  |  |  |  |  |  |
|  |  |  |  |  |  |  |  |
|  |  |  |  |  |  |  |  |
|  |  |  |  |  |  |  |  |
|  |  |  |  |  |  |  |  |
|  |  |  |  |  |  |  |  |
|  |  |  |  |  |  |  |  |
|  |  |  |  |  |  |  |  |
|  |  |  |  |  |  |  |  |
|  |  |  |  |  |  |  |  |
|  |  |  |  |  |  |  |  |
|  |  |  |  |  |  |  |  |
|  |  |  |  |  |  |  |  |
|  |  |  |  |  |  |  |  |
|  |  |  |  |  |  |  |  |
|  |  |  |  |  |  |  |  |
|  |  |  |  |  |  |  |  |
|  |  |  |  |  |  |  |  |
|  |  |  |  |  |  |  |  |

**Additional File 4-C: Results of Included Studies**

| Baseline  Hb (g/dl) | Baseline  Ferritin  (mcg/L) | Baseline  TSAT (%) | Hb change mean (g/dl) IV iron vs.  comparator | Hb change (% achieved target) | Transfusion  (%) IV iron v  comparator | Infection (%) | Mortality (%) | Length of Hospital Stay |
| --- | --- | --- | --- | --- | --- | --- | --- | --- |
|  |  |  |  |  |  |  |  |  |
|  |  |  |  |  |  |  |  |  |
|  |  |  |  |  |  |  |  |  |
|  |  |  |  |  |  |  |  |  |
|  |  |  |  |  |  |  |  |  |
|  |  |  |  |  |  |  |  |  |
|  |  |  |  |  |  |  |  |  |
|  |  |  |  |  |  |  |  |  |
|  |  |  |  |  |  |  |  |  |
|  |  |  |  |  |  |  |  |  |
|  |  |  |  |  |  |  |  |  |
|  |  |  |  |  |  |  |  |  |
|  |  |  |  |  |  |  |  |  |
|  |  |  |  |  |  |  |  |  |
|  |  |  |  |  |  |  |  |  |
|  |  |  |  |  |  |  |  |  |
|  |  |  |  |  |  |  |  |  |
|  |  |  |  |  |  |  |  |  |
|  |  |  |  |  |  |  |  |  |
