# Supplementary material for: Role of preoperative intravenous iron therapy to correct anemia before major surgery: study protocol for systematic review and meta-analysis
Source: Syst Rev. 2015 Mar 15;4:29. doi: 10.1186/s13643-015-0016-4 (PMC4369835; doi:10.1186/s13643-015-0016-4)
Supplement: Additional file 5: — Risk of bias detection. Cochrane risk of bias tool. [file 13643_2015_16_MOESM5_ESM.doc]

**Additional file 5: The Cochrane Collaboration’s tool for assessing risk of bias**

| **Source of bias** | **Review authors’ judgment** | **RCT 1** | **RCT 2** | **RCT 3** | **…** |
| --- | --- | --- | --- | --- | --- |
| Random sequence generation | Selection bias due to inadequate generation of a randomized sequence | low/unclear/high | low/unclear/high | low/unclear/high |  |
| Allocation concealment | Selection bias due to inadequate concealment of allocations before assignment | low/unclear/high | low/unclear/high | low/unclear/high |  |
| Blinding of participants and personnel | Performance bias due to knowledge of the allocated interventions by participants and personnel during the study | low/unclear/high | low/unclear/high | low/unclear/high |  |
| Blinding of outcome assessment | Detection bias due to knowledge of the allocated interventions by outcome assessment | low/unclear/high | low/unclear/high | low/unclear/high |  |
| Incomplete outcome data | Attrition bias due to amount, nature, or handling of incomplete outcome data | low/unclear/high | low/unclear/high | low/unclear/high |  |
| Selective reporting | Reporting bias due to selective outcome reporting | low/unclear/high | low/unclear/high | low/unclear/high |  |
| Other sources of bias | Bias due to problems not covered elsewhere in the table. | low/unclear/high | low/unclear/high | low/unclear/high |  |
