# Supplementary material for: Role of preoperative intravenous iron therapy to correct anemia before major surgery: study protocol for systematic review and meta-analysis
Source: Syst Rev. 2015 Mar 15;4:29. doi: 10.1186/s13643-015-0016-4 (PMC4369835; doi:10.1186/s13643-015-0016-4)
Supplement: Supplementary file 5 [file 13643_2015_16_MOESM3_ESM.docx]

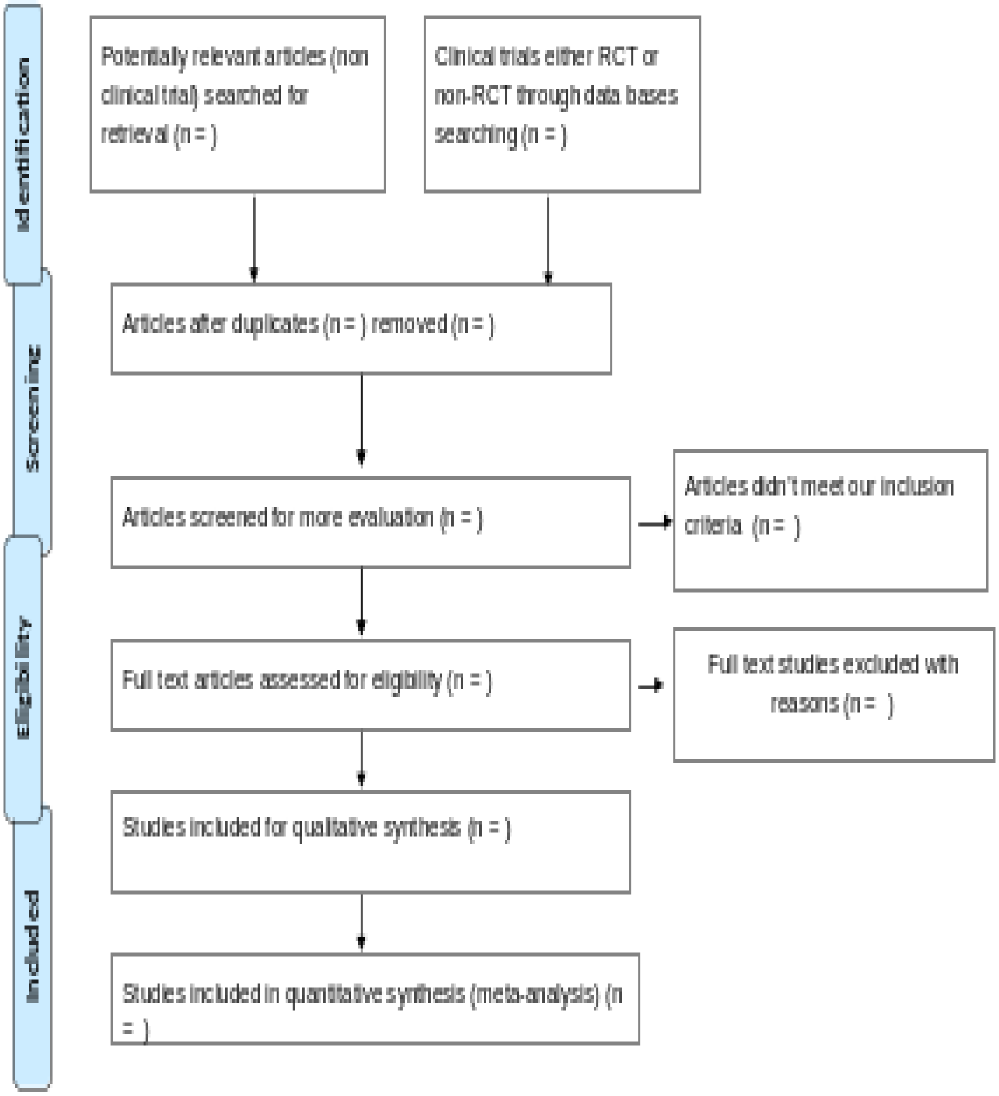
Identification
Screening
Eligibility
Included
Potentially relevant articles (non clinical trial) searched for retrieval (n =)
Clinical trials either RCT or non-RCT through data bases searching (n =)
Articles after duplicates (n =) removed (n =)
Articles screened for more evaluation (n =)
Articles didn't meet our inclusion criteria (n =)
Full text articles assessed for eligibility (n =)
Full text studies excluded with reasons (n =)
Studies included for qualitative synthesis (n =)
Studies included in quantitative synthesis (meta-analysis) (n =)
